# Clinical Impact of Lymphadenectomy after Neoadjuvant Chemotherapy in Advanced Epithelial Ovarian Cancer: A Review of Available Data

**DOI:** 10.3390/jcm10020334

**Published:** 2021-01-18

**Authors:** Stephanie Seidler, Meriem Koual, Guillaume Achen, Enrica Bentivegna, Laure Fournier, Nicolas Delanoy, Huyên-Thu Nguyen-Xuan, Anne-Sophie Bats, Henri Azaïs

**Affiliations:** 1 AP-HP.CUP, Service de Chirurgie Cancérologique Gynécologique et du Sein, Hôpital Européen Georges-Pompidou, 75015 Paris, France; seidler.steph@gmail.com (S.S.); meriem.koual@aphp.fr (M.K.); guillaume.achen@aphp.fr (G.A.); enrica.bentivegna@aphp.fr (E.B.); huyen_thu.nguyen_xuan@aphp.fr (H.-T.N.-X.); anne-sophie.bats@aphp.fr (A.-S.B.); 2 Swiss Medical Network, Clinique de Genolier, 1272 Genolier, Switzerland; 3 Faculté de Médecine Paris-Descartes, Université de Paris, 75006 Paris, France; laure.fournier@aphp.fr; 4 INSERM UMR-S 1124, Université de Paris, Centre Universitaire des Saints Pères, 75006 Paris, France; 5 AP-HP.CUP, Service de Radiologie, Hôpital Européen Georges-Pompidou, 75015 Paris, France; 6 AP-HP.CUP, Service D’oncologie Médicale, Hôpital Européen Georges-Pompidou, 75015 Paris, France; nicolas.delanoy@aphp.fr; 7 Centre de Recherche des Cordeliers, INSERM UMR-S 1138, 75006 Paris, France

**Keywords:** epithelial ovarian cancer, lymphadenectomy, gynecologic oncology

## Abstract

Recent robust data allow for omitting lymph node dissection for patients with advanced epithelial ovarian cancer (EOC) and without any suspicion of lymph node metastases, without compromising recurrence-free survival (RFS), nor overall survival (OS), in the setting of primary surgical treatment. Evidence supporting the same postulate for patients undergoing complete cytoreductive surgery after neoadjuvant chemotherapy (NACT) is lacking. Throughout a systematic literature review, the aim of our study was to evaluate the impact of lymph node dissection in patients undergoing surgery for advanced-stage EOC after NACT. A total of 1094 patients, included in six retrospective series, underwent either systematic, selective or no lymph node dissection. Only one study reveals a positive effect of lymphadenectomy on OS, and two on RFS. The four remaining series fail to demonstrate any beneficial effect on survival, neither for RFS nor OS. All of them highlight the higher peri- and post-operative complication rate associated with systematic lymph node dissection. Despite heterogeneity in the design of the studies included, there seems to be a trend showing no improvement on OS for systematic lymph node dissection in node negative patients. A well-conducted prospective trial is mandatory to evaluate this matter.

## 1. Introduction

The widely accepted aim of the surgical part of the treatment for advanced epithelial ovarian cancer (EOC) is to achieve macroscopic complete resection of peritoneal metastases. The very recent years have shown great improvement in the treatment of advanced EOC, and the main surgical step forward followed the publication of the results of the LION trial late 2018 [1]. This trial demonstrated the non-beneficial effect of lymph node dissection (LND) on overall survival (OS) and recurrence-free survival (RFS) during primary cytoreductive surgery (CRS) for stage IIb-IV EOC in the setting of clinically and radiologically node negative patients.

These data emphasize the idea that the burden of peritoneal involvement and the ability to reach a complete macroscopic CRS is the main prognosis factor regardless of lymph node removal. These facts have been shown in an upfront CRS setting. Nowadays, advanced EOC patients often undergo neoadjuvant chemotherapy (NACT), with an apparently equivalent oncologic outcome in comparison with primary CRS when complete macroscopic resection has been achieved [2]. The aim of this study was to review clinical data available, to assess the impact of lymph node dissection in patients undergoing surgery for advanced-stage EOC after NACT.

## 2. Method

Our literature review was conducted by using PRISMA criterion. PubMed, Cochrane Library and clinicaltrials.gov were searched until August 2020, without restriction regarding publication year. Keywords used were “lymphadenectomy”, “neo-adjuvant chemotherapy”, “advanced ovarian cancer”, “lymph node dissection”, “interval debulking surgery” and their MESH terms. Articles were screened by their titles, then by the abstracts and, finally, the whole text, if the article matched the criteria. Duplicates were excluded. Bibliography of selected articles were also checked, to verify the absence of omitted publications within the broad scope of our review. We included articles in English, German and French languages. Exclusion criteria were primary CRS, non-epithelial ovarian cancer, early stage ovarian cancer, and series where primary surgery and NACT patients were mixed and no separate results could be obtained.

We extracted data such as the study design, population characteristics, number of patients and information regarding their health status, tumoral and treatment details, and stage repartition according to the classification of the International Federation of Gynecology and Obstetrics (FIGO) [3]. The type of surgical procedure performed, number of resected lymph node and final pathological status were also included.

Our main objective was to evaluate the impact on OS and RFS. Secondary objectives were the surgical considerations of these procedures in terms of duration and peri- and post-operative complications. We also looked at radiologic examinations, when available, to evaluate if lymph node status could be predicted by imaging.

## 3. Results

### 3.1. Study Selection and Characteristics of the Population

**Figure 1:**
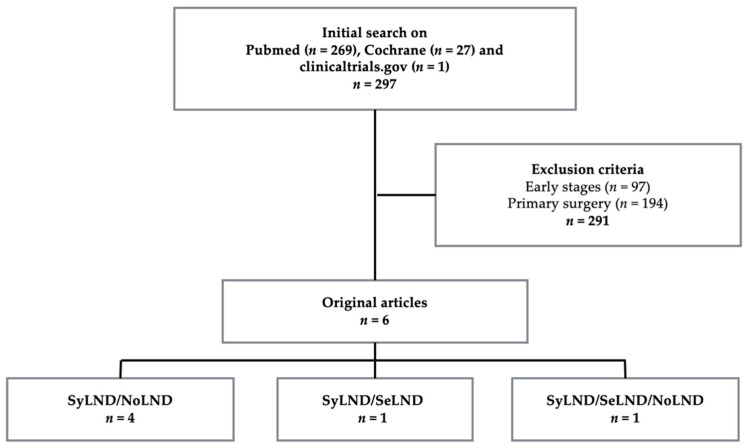
Study selection flowchart. SyLND = systematic lymph node dissection, SeLND = selective lymph node dissection, and NoLND = no lymph node dissection.

The study selection is illustrated in the flowchart below (Figure 1). We identified six articles that meet our criteria. All were retrospective series. Half of them were multi-centric. The studies’ characteristics are described in Table 1 [4,5,6,7,8,9]. Altogether, this review summarizes the outcomes for 1094 patients. Macroscopic complete CRS (classified CC-0) was achieved from 80% up to 100%, except for Eoh’s series [7] and the no lymphadenectomy group of Bund’s study [9], where the rate of complete CRS was respectively 35% and 50%.

Depending on the series, patients underwent different patterns of nodal surgery procedures:

- Systematic lymph node dissection (SyLND): systematic pelvic and para-aortic lymph node dissection up to the renal vein.
- Selective lymphadenectomy (SeLND): resection of bulky nodes only or resection of previously known positive lymph nodes, based on pre-operative imaging.
- No lymphadenectomy (NoLND): no lymph node dissection.

Of the 1094 included patients, 579 (52.7%) underwent SyLND, 213 (19.7%) SeLND only and 302 (27.6%) NoLND.

Regarding the type of NACT, carboplatin-paclitaxel regimen was the most widely used regimen (>80%), except for Iwase’s Japanese series, where it consisted in ifosfamide, etoposide, cyclophosphamide, doxorubicin and cisplatin half of the time. When information was available, BRCA-mutated patients were equally represented in the groups compared, but very little is reported regarding adjuvant treatments like olaparib or bevacizumab.

### 3.2. Main Outcomes

All the studies had a survival analysis as primary endpoint to evaluate the impact of lymph node dissection. Four studies compared SyLND to NoLND [4,5,6,9], one study compared SyLND to SeLND [7] and one compared the three groups [8].

#### 3.2.1. Lymph Node Status Assessment

The impact of lymph node dissection regarding suspected preoperative lymph node status was assessed in two studies [7,8].

Only one study mentioned details concerning the pre-operative lymph node status assessment: All patients included in Song and Gao’s study underwent CT, MRI or PET–CT, before and at the end of NACT. Positive lymph nodes were considered as greater than 15 mm on CT or MRI and/or hypermetabolic on PET–CT. Negativation was radiologically suspected when lymph nodes measured less than 10 mm on CT or MRI or presented no hypermetabolism on PET–CT. Among radiologically node negative patients, micrometastases were found in 25% of cases. No more information about the specificity and sensitivity of these tools is reported [8].

In the study of Eoh et al., OS was higher in the SyLND group than in patients undergoing SeLND, regardless of the suspected lymph node status on preoperative CT. On the other hand, RFS was not improved in the SyLND group, as compared to SeLND, in patients with positive nodes [7].

#### 3.2.2. Pathology Results

Details about the pathology results are summarized in Table 2. The number of lymph nodes removed was superior to 25 in SyLND groups and varied from 26 to 46. Bulky nodes resection concerned six to eight nodes. The percentage of metastatic lymph nodes ranged from 11% to 66%. In the SeLND (therefore clinically suspicious) group of Eoh’s series, it was up to 66% positive lymph nodes after NACT [7]. In Song’s series, among patients with no radiological evidence of lymph node involvement, 25% of micrometastases were still retrieved on pathological examination [8].

#### 3.2.3. Oncological Outcomes

Results are summarized in Table 3.

##### Recurrence-Free Survival

Two-year RFS was not found to be statistically different in NoLND versus SyLND groups in the Fagotti et al. study (respectively 25% and 36% (*p* = 0.834)) [4]. Schwartz et al. reported no different median RFS among those same groups, with respectively 9.7 months (NoLND) and 10.4 months (SyLND). The difference remained not statistically significant after adjustment for age, grade and peritoneal carcinomatosis index with HR = 1.43, 95% CI = 0.86–2.39 and *p* = 0.17 [6]. For Bund et al., the median RFS was 18.3 months for SyLND and 16.6 months for NoLND, a difference not statistically significant in either case [9]. Iwase et al. also showed similar two-year RFS in NoLND group SyLND subgroup with positive lymph nodes (*p* = 0.895). However, among patients who underwent SyLND, pathological negative lymph nodes status was associated with an improved two-year RFS (*p* = 0.002) [5].

Similarly, median RFS was not different in Eoh’s study (17 versus 12 months, *p* = 0.74, respectively, for SyLND versus SeLND), but, interestingly, the subgroup analysis among radiological node negative patients found an improved RFS in patients having underwent SyLND versus SeLND (*p* = 0.002).

Finally, Song et al. compared the three groups and found a median RFS of 22 months (NoLND), 28 months (SeLND) and 30.5 months (SyLND) (*p* = 0.049). NoLND appeared as an independent factor affecting RFS on the Cox analysis (HR = 1.729, 95% CI 1.213 to 2.464, *p* = 0.002), and there was no difference for RFS between SeLND and SyLND (HR = 1.097, 95% CI 0.815 to 1.478, *p* = 0.541) [8].

##### Overall Survival

Most studies did not show any effect of SyLND on OS: Among the four studies comparing SyLND to NoLND, Fagotti et al. report a two-year OS of 69% for SyLND and 88% for NoLND (*p* = 0.77) [4]; Schwartz et al. describe a median OS of 36.3 months (SyLND) and 33.1 months (NoLND) (*p* = 0.42), as well as a five-year OS rate at 35% for SyLND and 25.8% for NoLND. The difference was not statistically different after adjustment for age, grade and peritoneal carcinomatosis index, with HR =1.88, 95% CI 0.89–3.94; *p* = 0.088. The same trend is reported by Bund et al., with 26.8 months (SyLND) and 27.6 months (NoLND), (*p* = 0.73) [6,9].

Iwase et al. expressed their results according to gross residual disease: Two-year OS and five-years OS were, respectively, 88.8% and 43.5% in the no gross residual disease group, versus 40% and 0% in the optimal and suboptimal surgery groups (*p* = 0.001). According to lymph node status, the five-year OS was 62% for node negative patients, 26% for node positive patients and 19% for the NoLND group. There was no difference regarding five-year OS between SyLND patients with positive lymph nodes and NoLND patients (*p* = 0.61). On uni- and multivariate analyses, there were no differences on OS, whether lymph node dissection was performed or not, and even no differences if the patient had metastatic lymph node involvement or not. The only predictor of OS on multivariate analysis was gross residual disease [5]. Eoh et al.’s series is the only positive study on OS: 37 months (SyLND) and 28 months (SeLND), *p* = 0.001, regardless of the suspected lymph node status [8].

Finally, Song et al., comparing the three groups, report a median OS of 50 months (SeLND), 59 months (SyLND) and 57 months (NoLND); the five-year OS was, respectively, 47.1%, 47% and 46.2%, with no difference between the groups (*p* = 0.56) [8].

### 3.3. Secondary Outcomes

Results about secondary outcomes, like surgery duration, lymphocele formation and lower limbs lymphedema, were unanimous with statistically significant reduction in operative time, whether with SeLND or NoLND, compared to SyLND. The same trend was encountered for median blood loss and transfusion need; SeLND as well as NoLND showed significantly less lymphocele formation and lower limbs edema (*p* = 0.001) [5,6,8]. Overall, there were significantly less Clavien Dindo grade I and II complications when the lymphadenectomy was not performed [4]. Logically, Bund reported higher intra-operative (20.9% versus 6.5%, *p* = 0.003) and post-operative complications (26.7% versus 12.8%, *p* = 0.0001) for the SyLND group, as compared to the NoLND group [9].

## 4. Discussion

This review on the impact of lymph node dissection after NACT for advanced-stage EOC highlights the scarcity of scientific data on the subject: based on very few retrospective studies with different designs, making their results uneasy for comparison. Nevertheless, it enables us to underline that lymph node dissection’s benefits on OS and RFS are not so evident.

International recommendations for systematic lymph node dissection for advanced EOC have recently been adapted towards a de-escalation in the primary surgical treatment. The question of lymph node dissection for clinically node negative patients was answered through the LION trial [1]. The matter for initially suspicious lymph nodes who responded to NACT remains unanswered. These six studies, all retrospective, do not evaluate lymph node status by imaging, whether at diagnosis or after NACT treatment.

Nevertheless, this study highlights contradictory results regarding the impact on survival of lymph node dissection after NACT. Only one study reveals a positive effect on OS [7], and two on RFS [7,8]. The four remaining series fail to demonstrate any beneficial effect on survival, neither on OS nor RFS [4,5,6,9]. Of note, more patients in Eoh’s series were suspected to be node positive in the SyLND group and raising the issue that, for node positive patients, lymph node dissection might not catch up for the worse prognosis [7]. In Song’s series, the difference was between NoLND and SyLND, but not between SeLND and SyLND [8].

All series highlight the importance of complete macroscopic peritoneal cytoreduction and confirm that macroscopic residual disease is the main prognostic factor. It seems reasonable to think that the effect of lymph node dissection should be better assessed in case of no complete macroscopic CRS. We need to mention the fact that Eoh et al.’s series comprises only 36% of complete CRS, and the NoLND group of Bund and al.’s series reached 50% of complete CRS [7,9]. Their SyLND group and Iwase’s series reached, respectively, 88% and 80% of complete CRS, whereas the remaining studies included only patients who benefited from macroscopic complete CRS, and these found no effect of SyLND on OS [4,6,8].

Lymph node dissection type (systematic vs. selective) and its quality are issues that need to be addressed. Some series compare SyLND to SeLND or even to no dissection at all. A concern remains with the opening of the retroperitoneal spaces during SeLND and the morbidity of this dissection. The precise identification of the pre-chemotherapy clinically suspicious nodes could be questioned. Finally, a major concern is that the choice of dissection (or not) was not justified: we do not know why patients were assigned SeLND or NoLND. Was it because of the initial lymph node status, because of their response to chemotherapy or because of patients’ comorbidities. This matter of crucial clinical importance deserved to be developed.

### 4.1. Metastatic Lymph Node and Its Effect on Outcomes

The LION trial demonstrated that more than 50% of patients with radiological and clinical node judged negative had positive nodes on pathological analysis and that it did not changed the outcomes for advanced EOC whether removed or not [1]. The question if this postulate can be transposed to the NACT setting is not answered yet; however, it is frequently performed during clinical practice. The lymph node sanctuary of chemoresistance has been described for a long time, with the finding that lymph node metastasis persisted in 36% to 54% of lymph nodes after chemotherapy, suggesting that metastatic lymph nodes are not chemosensitive lesions [10]. Interesting data from Di Re et al. reported, in 2000, a 17% of positive lymph nodes after NACT [11]. Dell’Anna, in 2012, in a series of second-look surgery, showed 13% of lymph node metastasis in the case of no residual disease and 30% of lymph node metastasis if residual disease was present. In this series, SyLND did not improve RFS nor OS, nor influence the recurrence location [12]. In the six reviewed studies, the percentage of metastatic lymph nodes ranged from 24% to 66%. It seems that, for all but one study, occult metastasis did not impact OS.

### 4.2. Recurrence Occurrence and Location

Recurrence occurred in 70% to 80% of advanced EOC patients, except for the NoLND group of Fagotti’s series, where 62% of patients relapsed, but the difference was not statistically significant [4]. The same series also showed no difference between the two groups in the location of relapses.

Lymph node recurrence occurred in 6% to 20% in the SyLND groups, 11% and 16% in the SeLND groups and 29% to 32% in the two NoLND groups, when the information was available [4,5,7,8].

According to lymph node status (node negative, node positive and unknown lymph node status), patients suffered recurrences in the lymph nodes in respectively 25%, 26% and 35%, whereas the peritoneal recurrence rates were 82%, 76% and 70%; however, care must be taken, because all recurrence sites were counted, if multiple recurrence occurred [5].

Isolated lymph node relapses are said to be rare; in Eoh’s series, they occurred in 10% of SeLND group and 6% for the SyLND group [7]. In Iwase’s series, the realization of lymphadenectomy or not and the presence of lymph node metastases or not were both not associated with OS, whether on uni- or on multivariate analysis. On uni- and multivariate analysis, several authors found no residual tumor to be associated with improved RFS [7,8] and OS [5].

### 4.3. Pre-Operative Imaging

Since the omission of systematic lymph node dissection for clinically negative patients in primary surgery, a reliable diagnostic tool is mandatory for initial and preoperative assessment, also in the interval setting. Eoh reported respectively a 70% and 55% performance rate for CT and MRI, in line with the published series [7]. This year, Widschwendter et al. reported a negative predicted value of 58% and a positive predicted value of 80% for CT [13]. PET-CT shows a 77% to 83.8% sensitivity [14]. However, this must be balanced with the fact that some 25% of occult lymph node metastasis are found in clinically node negative patients. A recent meta-analysis of intra-operative lymph node assessment by clinical examination for patients undergoing primary surgery had a good positive predictive value if suspicious on examination, thus enabling physicians not to “undertreat” suspiciously positive patients after complete macroscopic CRS [15].

### 4.4. Studies Limitations and Interest of This Review

All the series included are retrospective. They were all based on different design, whether for the initial lymph node status, the dissection type or even the timing of surgery after three of six cycles of chemotherapy. In 80% to 100% of the cases, patients present a serous ovarian carcinoma. This is a distribution of histological subtypes among the patient’s population consistent with what is usually observed. Nevertheless, given all of these considerations, this review is the first to synthesize the available data regarding lymph node dissection in advanced EOC after NACT. In the studies by Fagotti, Iwase, Bund, Schwartz and Song, only the rates of patients with serous tumors are reported. The other histological subtypes are not detailed. The only author who provides details of the histological subtypes is Eoh. It would be interesting to complete these analyses on larger patient populations, to study the less frequent histological subtypes and their prognosis (RFS and OS) according to the achievement or not of systematic lymph node dissection.

## 5. Conclusions

The LION trial, in the primary surgery setting, enabled a de-escalation in the indications for lymph node dissection for node negative patients, by demonstrating that, whether performed or not, lymph node dissection did not have any impact on OS. This question is also relevant in the NACT setting, where prognosis depends on peritoneal disease burden and where patients, by definition, have extensive peritoneal disease. Despite some heterogeneity in the studies reviewed here, there seems to be the same trend as in the primary surgery, meaning that lymph node dissection may not be associated with improved survival for node negative patients. A proper prospective study should, thus, evaluate this matter.

## Figures and Tables

**Table 1:** Studies’ characteristics.

Authors	Type of LND	Sample Size	Age (Years-Median) or *n* (%)	FIGO Stage *n* (%)	Histopathologic Type *n* (%)	Median Number NACT Cycles	Residual Tumor (cm) *n* (%)
Fagotti [4]	NoLND (9 SeLND)	101	62.0	III: 81 (80.2) IV:20 (19.8)	Serous: 99 (98.0) Others: 2 (2.0)	4	0: 81 (80.2) <1: 20 (19.8)
	SyLND	50	62.0	III: 37 (74.0) IV:13 (26.0)	Serous: 47 (94.0) Others: 3 (6.0)	6	0: 41 (82.0) <1: 9 (18.0)
Iwase [5]	NoLND SyLND	124 (38 NoLND/86 SyLND)	58.0	III: 83 (66.9) IV:41 (33.1)	Serous: 105 (85.0) Others: 19 (15.0)	6	0: 98 (79.0) <1: 15 (12.1) ≥1: 11 (8.9)
Schwartz [6]	NoLND	47	≤55: 14 (29.8) 56–69: 22 (46.8) ≥71: 11 (23.4)	III: 27 (57.4) IV: 20 (42.6)	Serous: 38 (82.6) Others: 9 (17.4)	6	0: 47 (100.0)
	SyLND	54	≤55: 22 (40.8) 56–69: 24 (44.4) ≥71: 8 (14.8)	III: 43 (79.6) IV:11 (20.4)	Serous: 47 (87.0) Others: 7 (13.0)	5	0: 54 (100.0)
Eoh [7]	SeLND	68	60.5	III: 27 (39.7) IV: 41 (60.3)	Serous: 62 (91.2) Mucinous: 1 (1.5) Endometrioid: 3 (4.4) Clear cells: 2 (2.9)	3	0: 25 (36.8) <1: 43 (63.2)
	SyLND	65	53.8	III: 33 (50.8) IV: 32 (49.2)	Serous: 56 (86.2) Mucinous: 0 (0) Endometrioid: 3 (4.6) Clear cells: 4 (6.2) Others: 2 (3.0)	3	0: 22 (33.8) <1: 43 (66.2)
Song [8]	NoLND	67	56.0	III: 52 (77.6) IV: 15 (22.4)	Serous: 54 (80.6) Others: 13 (19.4)	3	0: 67 (100.0)
	SeLND	145	54.0	III: 108 (74.5) IV: 37 (25.5)	Serous: 124 (85.5) Others: 21 (14.5)	3	0: 145 (100.0)
	SyLND	118	55.5	III: 95 (80.5) IV: 23 (19.5)	Serous: 105 (89.0) Others: 13 (11.0)	3	0: 118 (100.0)
Bund [9]	NoLND	100	67.5	III: 78 (78.0) IV: 22 (22.0)	Serous: 100 (100.0)	NA	0: 50 (50.0) ≤0.25: 23 (23.0) >0.25: 27 (27.0)
	SyLND	155	59.0	III: 127 (81.9) IV: 28 (18.1)	Serous: 100 (100.0)	NA	0: 137 (88.4) ≤0.25: 9 (5.8) >0.25: 9 (5.8)

LND = lymph node dissection, SyLND = systematic lymph node dissection, SeLND = selective lymph node dissection, NoLND = no lymph node dissection, NACT = neoadjuvant chemotherapy, and NA = not available.

**Table 2:** Pathological lymph node analysis.

Authors	Number of Lymph Nodes Retrieved	Metastatic Lymph Node Rate on Pathological Examination	Overall Recurrence Rate	Lymph Node Recurrence Rate
Fagotti [4]	38 (SyLND)	28% (SyLND) 33% (NoLND)	70% (SyLND) 62% (NoLND)	no difference
Iwase [5]	46 (SyLND)	56%	81%	20% (SyLND) 29% (NoLND)
Schwartz [6]	26 (SyLND)	41%	NA	NA
Eoh [7]	27 (SyLND)/10 (SeLND)	66% (SeLND) 54% (SyLND)	80%	11% (SeLND) 6% (SyLND)
Song [8]	31 (SyLND)/8 (SeLND)	24%	70%	16% (SeLND) 13% (SyLND) 32% (NoLND)
Bund [9]	28 (SyLND)	11%	NA	no difference

SyLND = systematic lymph node dissection, SeLND = selective lymph node dissection, NoLND = no lymph node dissection, and NA = not available.

**Table 3:** Oncological outcomes.

Authors	RFS (Months)		OS (Months)	
Fagotti [4]	2y RFS: 36% (SyLND) 25% (NoLND)	*p* = 0.834	2y OS: 69% (SyLND) 88% (NoLND)	*p* = 0.77
Iwase [5]	2y RFS: 56% (SyLND N-) 24% (SyLND N+) 26% (NoLND)	*p* = 0.534	5y OS: 62% (SyLND N-) 26% (SyLND N+) 19% (NoLND)	*p* = 0.97
Schwartz [6]	10.4 (SyLND) 9.7 (NoLND)	*p* = 0.80	33 (SyLND) 35 (NoLND)	*p* = 0.17
Eoh [7]	12 (SeLND) 17 (SyLND)	*p* = 0.74	28 (SeLND) 37 (SyLND)	*p* = 0.001
Song [8]	28 (SeLND) 30 (SyLND) 22 (NoLND)	*p* = 0.566	50 (SeLND) 59 (SyLND) 57 (NoLND)	*p* = 0.049
Bund [9]	18.2 (SyLND) 16.6 (NoLND)	*p* = 0.70	26.8 (SyLND) 27.8 (NoLND)	*p* = 0.48

RFS = recurrence-free survival, OS = overall survival, SyLND = systematic lymph node dissection, SeLND = selective lymph node dissection, and NoLND = no lymph node dissection.

## Data Availability

Data is contained within articles cited in the manuscript.
